# DDX10 Exacerbates Exosomal PD-L1-Dependent T Cell Exhaustion via Phase Separation of Rab27b in Oral Squamous Cell Carcinoma

**DOI:** 10.34133/research.0697

**Published:** 2025-05-09

**Authors:** Bowen Li, Hao Cui, Wei Liu, Zhou Lan, Chang Liu, Yumiao Yang, Yuyue Zhao, Zhen Tian, Hao Chen, Guangtao Yu

**Affiliations:** Stomatological Hospital, School of Stomatology, Southern Medical University, Guangzhou, Guangdong 510280, China.

## Abstract

DEAD-box ATPase 10 (DDX10), a prominent RNA-binding protein in the DDX family, has a critical function in cancer progression. Nevertheless, its well-defined mechanisms in oral squamous cell carcinoma (OSCC) are still not well understood. Here, we identify that DDX10 is substantially increased in OSCC, which is positively correlated with poor prognosis and malignant behavior. Mechanistically, we found that DDX10 had physical interaction with Rab27b by undergoing phase separation. Knockdown of DDX10 inhibited Rab27b-mediated exosome secretion and the expression of programmed cell death-ligand 1 (PD-L1) within its contents. Furthermore, knocking down DDX10 could restore the function and infiltration of T cells, hence inhibiting the progression of OSCC. These findings highlight that the oncogenic role of DDX10 in promoting exosomal PD-L1 secretion via phase separation with Rab27b has been preliminarily validated in T cell exhaustion in OSCC. A potential strategy for improving OSCC immunotherapy may involve the inhibition of DDX10.

## Introduction

Oral squamous cell carcinoma (OSCC) is a prevalent form of malignancy in the head and neck region [1]. Projections suggest a 40% increase in the incidence of OSCC by 2040, along with a corresponding rise in mortality rates [2]. Current therapeutic strategies for OSCC include surgical resection, radiation therapy, chemotherapy, and immunotherapy; however, the long-term prognosis for individuals diagnosed with OSCC continues to be unsatisfactory, with a persistently low 5-year survival rate [3]. Therefore, the identification and validation of innovative treatment strategies and novel therapeutic targets for OSCC represent an urgent imperative.

Recently, the marked role of DEAD-box ATPase (DDX) protein family in the pathogenesis of tumor progression has garnered considerable attention, which is involved in tumor growth and metastasis [4–8]. The studies indicate that the DDX protein family has emerged as promising biomarker for the diagnosis, prognosis assessment, and pharmacological treatment of various malignancies including hepatocellular carcinoma (HCC), colorectal cancer, osteosarcoma, and breast cancer [9–12]. Specifically, the member DDX10 has been demonstrated to enhance the proliferation and metastasis of lung, colorectal, and ovarian cancers and it represents a potential therapeutic target of osteosarcoma and glioblastoma [13–16]. Nevertheless, the specific function of DDX10 in the pathogenesis and progression of OSCC warrants additional research to fully elucidate its importance.

Tumor immune evasion constitutes an important process during tumorigenesis and development. One typical factor is programmed cell death-ligand 1 (PD-L1)/programmed cell death-receptor 1 (PD-1) immune checkpoints, as classic immune inhibitory signaling pathways, which have been verified to exacerbate T cell functional exhaustion in OSCC [17–19]. Currently, the DDX protein family has been demonstrated to play a marked role in PD-L1/PD-1 immune checkpoint and tumor immune evasion. DDX6 was validated to enhance immune surveillance by facilitating the degradation of PD-L1 in clear cell renal cell carcinoma [20], and DDX5 has been shown to promote T cell infiltration, thereby inhibiting the progression of tongue cancer [21]. Moreover, the study has verified that DDX1 inhibits immune T cell function by up-regulating PD-L1 expression in HCC, and overexpression of DDX3X leads to immune suppression in melanoma by competitively enhancing PD-L1 expression [22]. Furthermore, the DDX protein family has been shown to have the potential for phase separation due to its intrinsically disordered regions (IDRs), which enable multivalent weak interactions that drive phase separation [23]. Phase separation, a type of molecular interaction, has a pivotal role in immune signal regulation and occurrence of tumors, and mutant phase separation promotes tumor progression by maintaining PD-1/PD-L1 checkpoint [24,25]. However, the function of DDX10 within the immune microenvironment of OSCC has yet to be investigated.

In this paper, we discovered that DDX10 can bind to Rab27b (exosome regulatory protein [26]) to form liquid–liquid phase separation condensates, thereby mediating T cell dysfunction through exosome-derived PD-L1 in OSCC. The TCGA database revealed that DDX10 is overexpressed in OSCC tissues, which correlates with increased malignancy and a poorer prognosis. RNA sequencing (RNA-Seq) analysis and further confirmatory molecular biology experiments identified that inhibition of DDX10 reduced the expression of Rab27b and exosomal PD-L1. Furthermore, our findings validated the function of DDX10 in promoting tumor-derived exosome expulsion and inducing T cell exhaustion. Mechanistically, the results demonstrated that DDX10 has physical interaction with Rab27b by undergoing phase separation. Knockdown of DDX10 inhibited Rab27b-mediated exosome secretion and the expression of PD-L1 within its contents. Tissue microarray analysis further revealed that DDX10 exacerbates poor prognosis in OSCC patients by inhibiting T cell infiltration. Taken together, DDX10 plays a pivotal role in cancer development and immune evasion, positioning it as a potential therapeutic target for cancer treatment.

## Results

### Overexpression of DDX10 in OSCC is associated with a poor prognosis

**Fig. 1.**
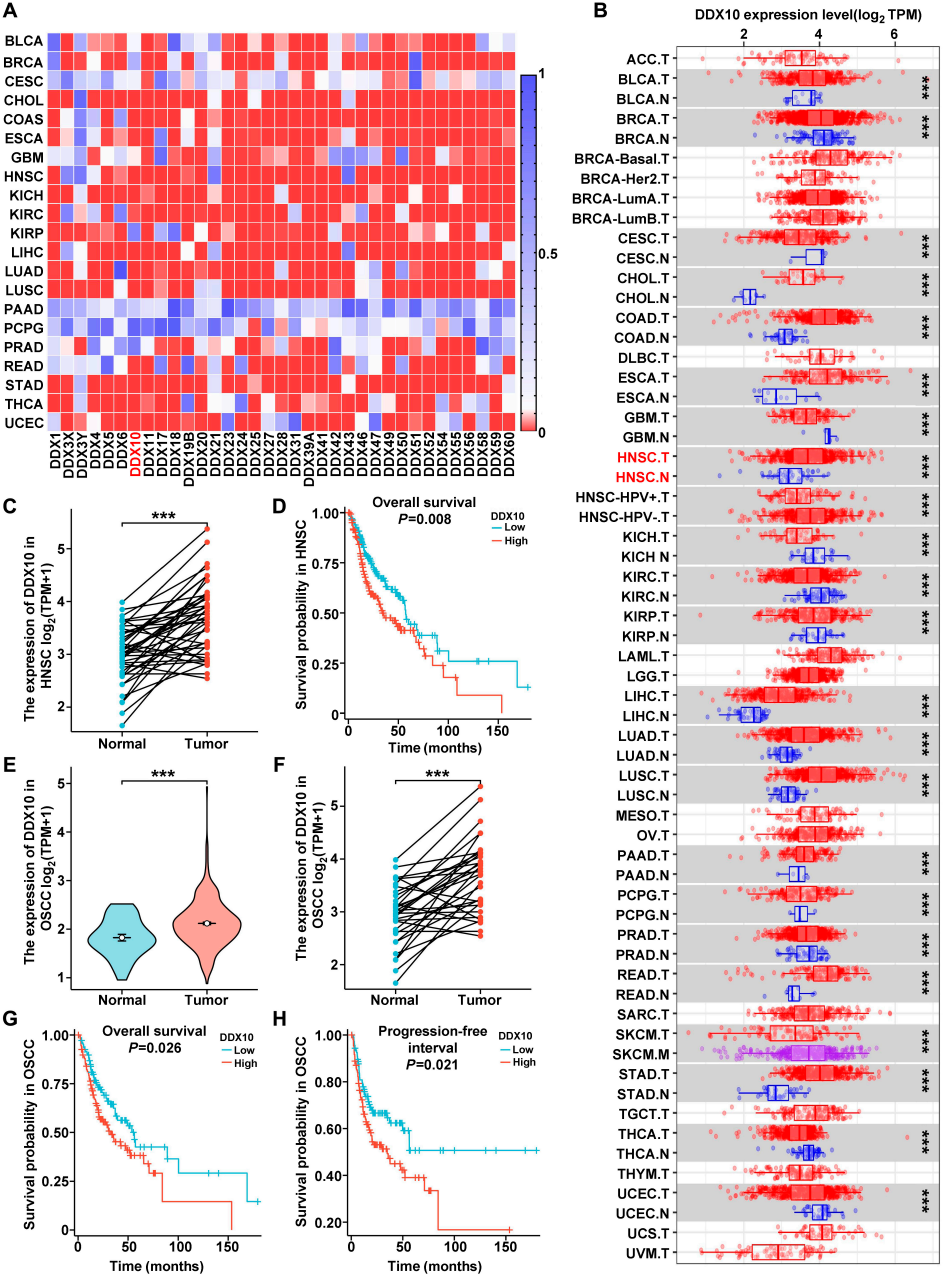
Overexpression of DDX10 in OSCC is associated with a poor prognosis. (A) Heatmap analysis from TCGA for expression of DDX family members in different tumors (red: *P* < 0.05, blue: *P* > 0.05). (B) Differences in expression of DDX10 in normal tissues and tumor tissues in TCGA datasets. (C) Analysis of DDX10 expression in HNSC samples and matched normal tissues in the TCGA cohort. (D) Overall survival analysis of HNSC patients stratified by DDX10 expression levels. (E) Analysis of DDX10 expression in oral tissues from OSCC patients and matched healthy controls utilizing the TCGA dataset. (F) Evaluation of DDX10 expression levels in matched tissue samples utilizing the TCGA database. (G) Overall survival curve of OSCC patients stratified by DDX10 expression levels. (H) Progression-free interval curve of OSCC patients stratified by DDX10 expression levels. ns: not significant, *P < 0.05, **P < 0.01, ***P < 0.001, ****P < 0.0001.

To explore the important role of DDX10 in the progression of OSCC, the TCGA database was utilized for a clinical prognostic analysis. As illustrated in Fig. 1A, the expression of DDX family proteins was examined across various cancers, and DDX10 is generally highly expressed in pan-cancer, and this trend is also observed in head and neck squamous cell carcinoma (HNSC). Specifically, our analysis revealed that DDX10 exhibits marked overexpression in HNSC tissues (Fig. 1B and C) and contributes to a poor prognosis (Fig. 1D). A comparable phenomenon was also observed in OSCC (Fig. 1E to H). Further analysis of the clinical characteristics of DDX10 expression in OSCC patients was further investigated. As illustrated in Table, elevated DDX10 expression is associated with more advanced tumor progression, typically indicating a less favorable prognosis. These results indicate that DDX10 is correlated with an unfavorable prognosis in OSCC and potentially functions as a valuable diagnostic marker and treatment target.

**Table.** Clinical features of the expression of DDX10 in OSCC patients by chi-square test

Characteristics	Low expression of DDX10	High expression of DDX10	*P* value
*n*	165	165	
Pathologic T stage, *n* (%)			<0.01
T1	21 (6.9%)	8 (2.6%)	
T2	55 (18%)	45 (14.8%)	
T3	28 (9.2%)	38 (12.5%)	
T4	45 (14.8%)	65 (21.3%)	
Pathologic N stage, *n* (%)			0.99
N0	58 (21%)	60 (21.7%)	
N1	24 (8.7%)	26 (9.4%)	
N2 and N3	53 (19.2%)	55 (19.9%)	
Clinical T stage, *n* (%)			<0.01
T1	14 (4.4%)	4 (1.2%)	
T2	64 (20%)	42 (13.1%)	
T3	41 (12.8%)	41 (12.8%)	
T4	45 (14.1%)	69 (21.6%)	
Clinical N stage, *n* (%)			0.81
N0	89 (28.2%)	80 (25.3%)	
N1	29 (9.2%)	27 (8.5%)	
N2	42 (13.3%)	46 (14.6%)	
N3	1 (0.3%)	2 (0.6%)	
Clinical stage, *n* (%)			<0.01
Stage I	10 (3.1%)	1 (0.3%)	
Stage II	46 (14.4%)	34 (10.6%)	
Stage III	34 (10.6%)	31 (9.7%)	
Stage IV	74 (23.1%)	90 (28.1%)	
Gender, *n* (%)			0.23
Female	56 (17%)	46 (13.9%)	
Male	109 (33%)	119 (36.1%)	
Age, *n* (%)			0.87
≤60	77 (23.4%)	79 (24%)	
>60	87 (26.4%)	86 (26.1%)	
Smoker, *n* (%)			0.15
No	50 (15.4%)	38 (11.7%)	
Yes	113 (34.9%)	123 (38%)	

### DDX10 promoted the malignant behavior of OSCC in vitro

**Fig. 2.**
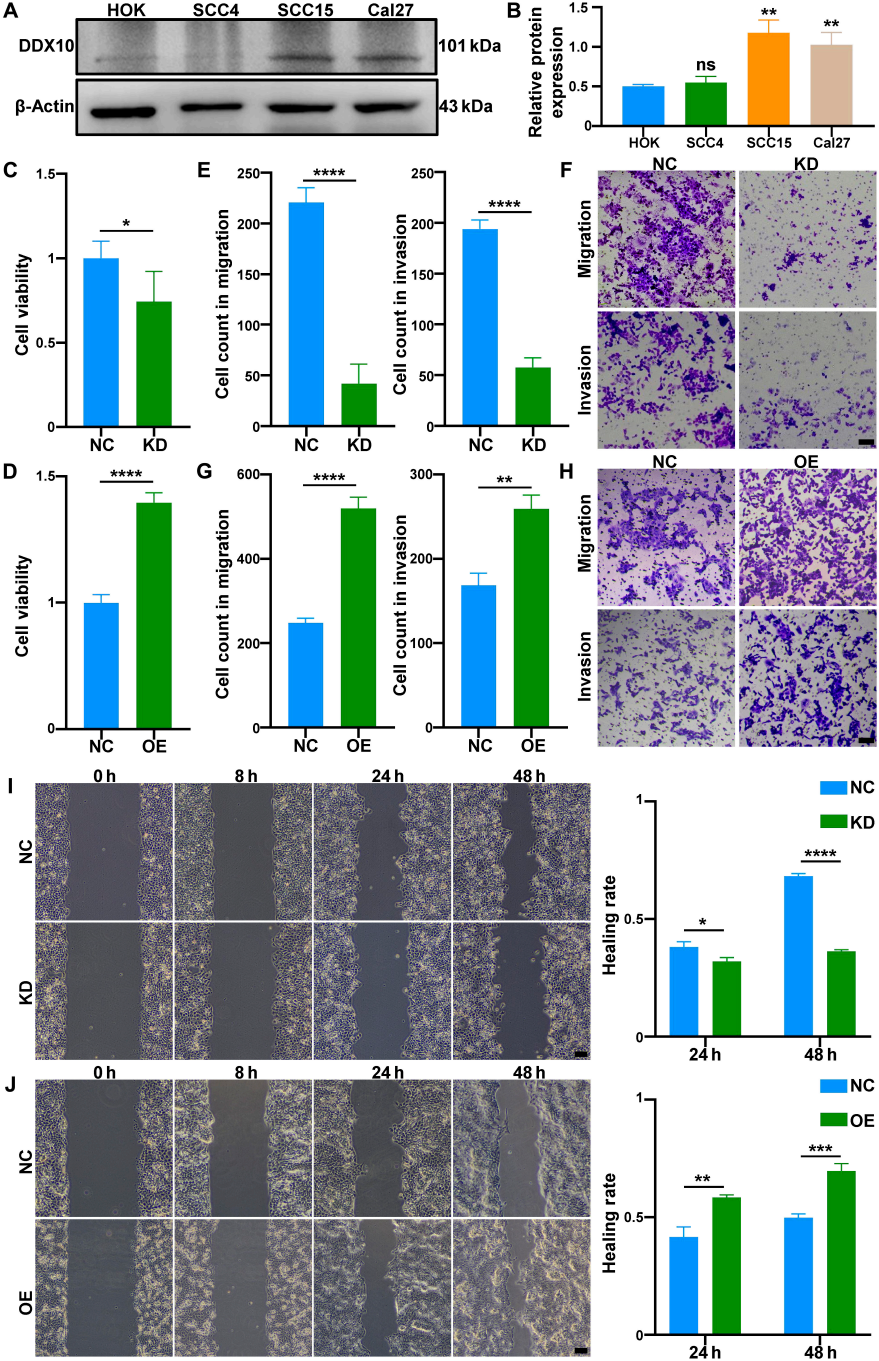
DDX10 promoted the malignant behavior of OSCC in vitro. (A and B) The expression levels of DDX10 were compared between normal oral epithelial cells (HOKs) and OSCC cells by Western blot assay. (C and D) Impact of DDX10 knockdown and overexpression on the proliferative capacity of SCC15 cells by CCK-8 assay in 24 h. (E to H) Impact of DDX10 knockdown and overexpression on the migratory and invasive capacity of SCC15 cells by Transwell assay. Scale bar, 100 μm. (I and J) Impact of DDX10 knockdown and overexpression on the migratory ability of SCC15 cells by wound healing assay. Scale bar, 200 μm. Results are expressed as the mean ± SD from a minimum of 3 experimental replicates. ns: not significant, *P < 0.05, **P < 0.01, ***P < 0.001, ****P < 0.0001.

To further elucidate the functional significance of DDX10 in OSCC, the expression levels of DDX10 were compared between normal oral epithelial cells (HOKs) and OSCC cells. The results demonstrated that DDX10 expression was notably elevated in SCC15 (**s**quamous carcinoma cells 15) and CAL27, particularly in SCC15 (Fig. 2A and B). Therefore, we selected SCC15 for subsequent experiments. Subsequently, we achieved knockdown of DDX10 using liposome-delivered siDDX10 and overexpression of DDX10 using plasmid-mediated delivery in SCC15 cells. Immunofluorescence (IF) staining and Western blotting (WB) analyses indicated the transfection efficiency and effect (Fig. S1A to D). Subsequently, we investigated the impact of DDX10 on the proliferative, migratory, and invasive abilities of SCC15 cells. DDX10 knockdown markedly inhibited the proliferative abilities of SCC15 cells compared to the control group in Cell Counting Kit-8 (CCK-8) assay, while overexpression of DDX10 significantly expedited the SCC15 cell proliferation rate in different time points (Fig. 2C and D and Fig. S1E and F). Moreover, Transwell assay demonstrated that the cell count in the knockdown (KD) group was markedly fewer than that in the normal control group (Fig. 2E and F); conversely, the cell count in the overexpression (OE) group was significantly higher than that in the control group (Fig. 2G and H). Similarly, the wound healing rate showed significant reduction in the KD group and promotion in the OE group relative to the negative control (NC) group (Fig. 2I and J), providing additional evidence that cell migration and invasion were significantly regulated by DDX10. In conclusion, these results collectively suggest that DDX10 markedly facilitates the proliferative, migratory, and invasive capabilities of OSCC.

### The biological mechanisms underlying DDX10 promoted the expression of Rab27b and PD-L1 while inducing T cell exhaustion

**Fig. 3.**
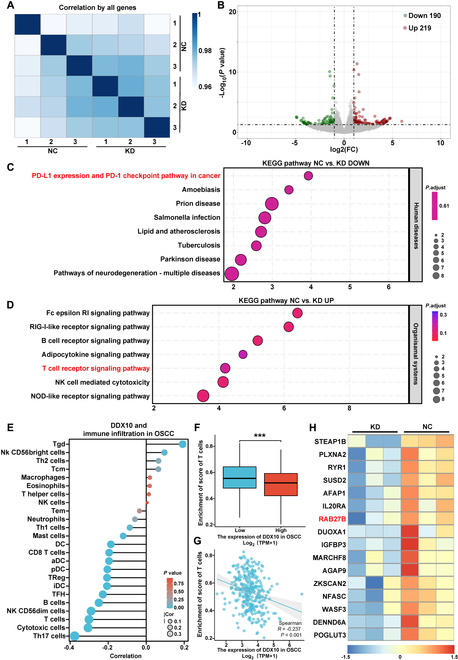
The biological mechanisms underlying of DDX10 promoted the expression of Rab27b and PD-L1 while inducing T cell exhaustion. (A) Correlation test in RNA-Seq specimens. (B) Volcano plot displayed the differential expressed genes (DEGs) between the KD group and the NC group. (C and D) KEGG analysis between the KD group and the NC group. (E) Bar chart from TCGA datasets shows the relationship between DDX10 and immune infiltration in OSCC. (F) TCGA cohort analyses of the T cell infiltration in OSCC with low and high DDX10 expression. (G) Scatterplot depicting that the correlation between DDX10 and T cell infiltration in the TCGA-OSCC dataset was determined using Pearson’s correlation coefficient. (H) Heatmap of the top genes among down-regulated genes from RNA-Seq data. Results are expressed as the mean ± SD from a minimum of 3 experimental replicates. ***P < 0.001.

In order to clarify the specific molecular mechanisms through which DDX10 mediates the progression of OSCC, we performed RNA-Seq analysis on SCC15 with or without knockdown of DDX10. Correlation analysis revealed consistency within groups and variations in gene expression profiles between experimental and control conditions (Fig. 3A). The volcano plot generated from the gene differential expression analysis indicates that the KD group contains a total of 219 up-regulated and 190 down-regulated (Fig. 3B). Pathway enrichment evaluation using Kyoto Encyclopedia of Genes and Genomes (KEGG) revealed that down-regulated genes in the knockdown cohort were significantly associated with the PD-L1/PD-1 immune checkpoint axis in cancer. (Fig. 3C). Additionally, the genes up-regulated in the KD group were enriched in the T cell receptor signal pathway (Fig. 3D). The PD-1/PD-L1 signaling pathway, a well-established immune checkpoint pathway, plays a crucial role in inhibiting T cell activity and facilitating tumor cell immune evasion. Our finding revealed that DDX10 expression in OSCC is negatively correlated with T cell infiltration, aligning with the analysis of the TCGA database (Fig. 3E to G). Based on these results, we tentatively propose that DDX10 might aggravate T cell exhaustion by up-regulating the PD-L1 expression and PD-1 checkpoint pathway in cancer, thereby facilitating tumor progression in OSCC. Furthermore, we conducted an in-depth investigation into the key differentially expressed genes as illustrated in Fig. 3H. The result showed that the expression of Rab27b was down-regulated upon knockdown of DDX10. Previous research has demonstrated that Rab27b is a crucial protein regulating exosome secretion and it facilitates the immune evasion of tumor cells [26,27]. Moreover, the study identified that tumor cells can secrete exosomes containing PD-L1 to target immune T cells, thereby promoting T cell exhaustion [28]. Consequently, we propose that DDX10 promotes Rab27b-dependent exosomal PD-L1 to exacerbate T cell exhaustion in OSCC.

### The interaction between DDX10 and Rab27b induced the secretion of exosome-derived PD-L1 in OSCC

**Fig. 4.**
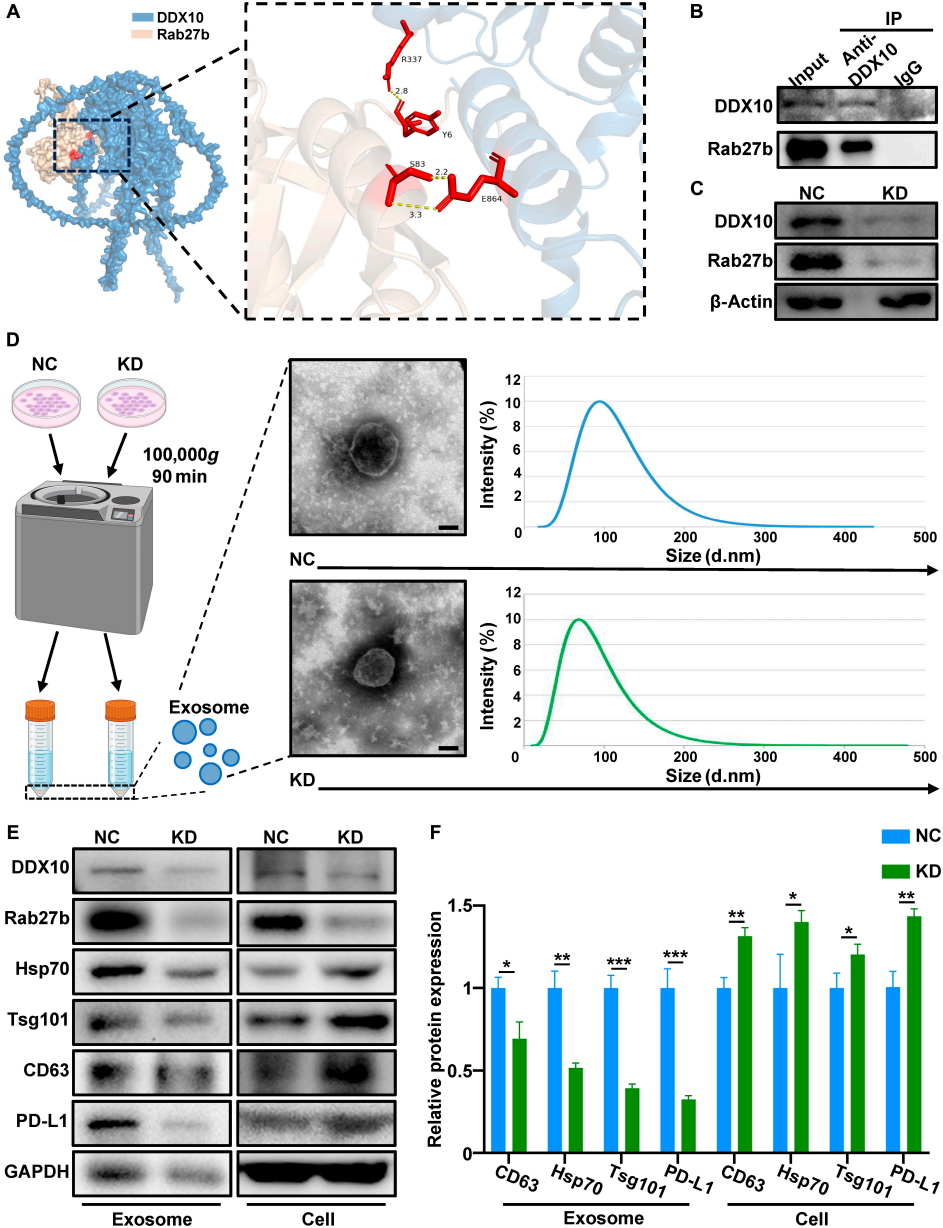
The interaction between DDX10 and Rab27b results in the secretion of exosome-derived PD-L1 in OSCC. (A) AlphaFold3 predicts the interaction between DDX10 and Rab27b based on their respective 3-dimensional protein structures. (B) The interaction of endogenous DDX10 with Rab27b in SCC15 cells was validated by Co-IP. (C) Protein levels of DDX10 and Rab27b were detected by WB assay in SCC15 cells with knockdown of DDX10. (D) TEM and dynamic light scattering analysis verified purified exosomes from the culture supernatant of SCC15 cells with knockdown of DDX10. Scale bar, 50 nm. (E and F) Protein levels of DDX10, Rab27b, PD-L1, and exosomal markers detected by WB assay in SCC15 cells and exosomes with or without DDX10 knockdown. Results are expressed as the mean ± SD from a minimum of 3 experimental replicates. *P < 0.05, **P < 0.01, ***P < 0.001.

To elucidate the regulatory effect of DDX10 on Rab27b, we initially verified whether DDX10 and Rab27b are physically associated. We postulated that DDX10 and Rab27b might potentially engage in mutual interaction by employing AlphaFold3 for protein docking (Fig. 4A). To validate the interaction between DDX10 and Rab27b, we conducted coimmunoprecipitation (Co-IP) and WB experiments. The experimental data substantiated the intrinsic interaction between DDX10 and Rab27b (Fig. 4B). After the knockdown of DDX10, it was determined that the expression level of Rab27b protein was likewise reduced through the WB experiment (Fig. 4C), which followed the previous sequencing outcomes. Furthermore, to illuminate the impact of knocking down DDX10 on Rab27b-mediated exosome secretion, we purified exosomes from the culture supernatant of SCC15 and subsequently characterized through transmission electron microscopy (TEM) and nanoparticle size analysis. The findings revealed that the size of the isolated exosomes varied between 30 and 200 nm, and the knockdown of DDX10 did not alter the morphology of the exosomes (Fig. 4D). Then, bicinchoninic acid assay (BCA) and WB experiments were performed on the extracted exosomes. The BCA assay indicated that the exosomal protein concentration in the KD group was markedly reduced compared to the NC group, confirming that knockdown of DDX10 decreased the total protein content of SCC15 exosomes (Fig. S2A); the corresponding WB results indicated that upon knocking down DDX10, the expression of glyceraldehyde-3-phosphate dehydrogenase (GAPDH) in exosomes was down-regulated (Fig. 4E). Meanwhile, the expression of exosome markers Hsp70, Tsg101, and CD63 within exosomes was decreased, while that in cells was augmented (Fig. 4E and F), suggesting that knockdown of DDX10 inhibited the secretion of exosomes regulated by Rab27b. To validate the previous sequencing results, we detected that the expression of exosomal PD-L1 was reduced after knocking down DDX10, while the content of PD-L1 in cells increased (Fig. 4E and F). These results indicate that knockdown of DDX10 inhibited Rab27b-mediated exosome secretion and the expression of PD-L1 contained therein.

### DDX10 undergoes phase separation to interact with Rab27b in vitro

**Fig. 5.**
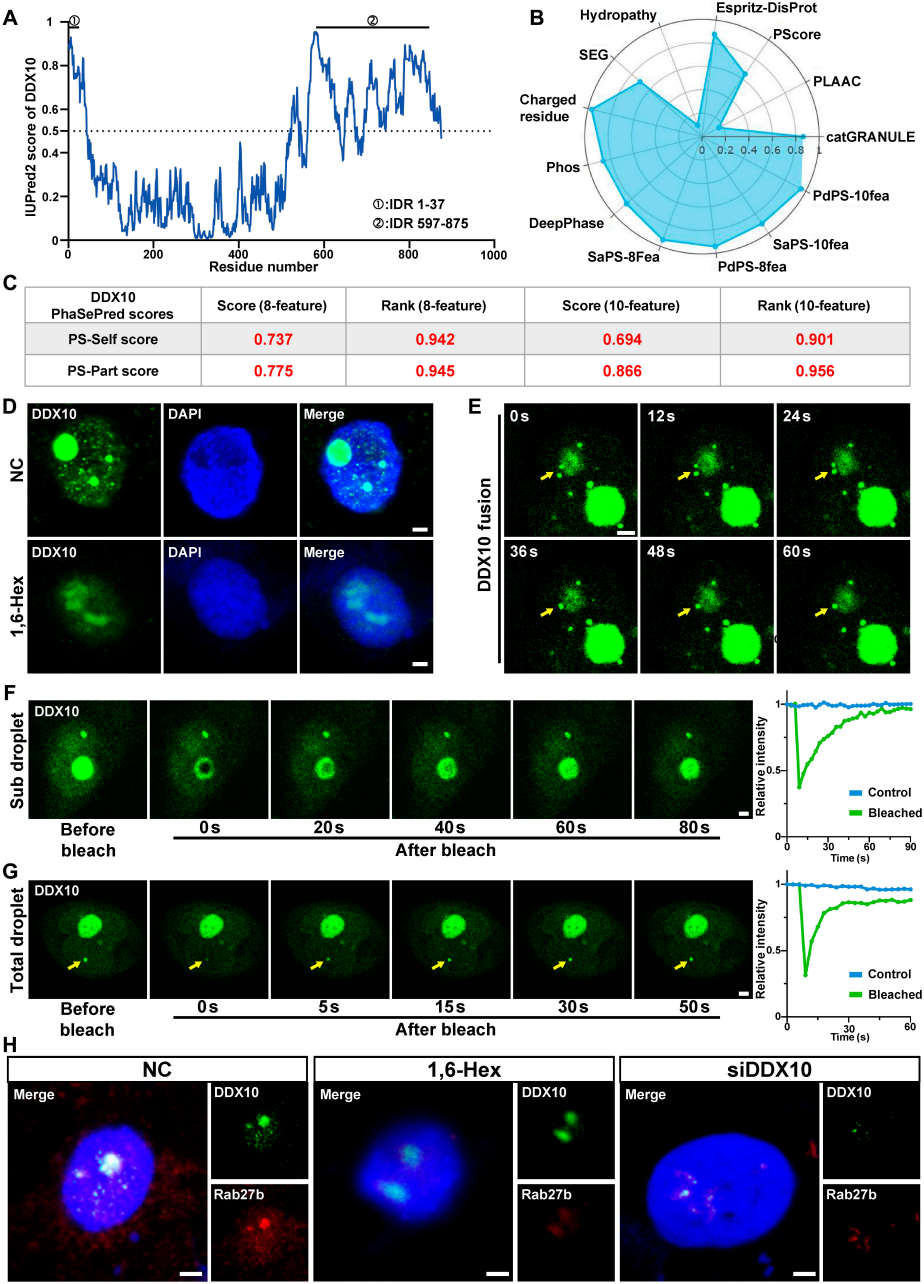
DDX10 undergoes phase separation to interact with Rab27b in vitro. (A) Intrinsically disordered tendency of DDX10 via iupred2a (IUP) reassigned disordered trend scores and phase separation prediction. (B and C) Self-phase separation and part-phase separation score from the Phase Separation Prediction website. (D) Representative micrographs of DDX10 analyzed by IF in SCC15 cells with and without 3% 1,6-Hex treatment. Scale bar, 2 μm. (E) Live-cell images showing the fusion of smaller droplets into larger spherical granules in DDX10-GFP-transfected SCC15 cells. Scale bar, 2 μm. (F and G) Live-cell images showing subregion (top) and total-region (bottom) FRAP of DDX10-GFP during the phase separation process in transfected SCC15 cells and quantification analysis of relative fluorescence intensity recovery. Scale bar, 2 μm. (H) Representative micrographs of colocalization of DDX10 and Rab27b analyzed by IF in SCC15 cells treated with PBS, 3% 1,6-Hex, and knockdown of DDX10. Scale bar, 2 μm.

To further explore the binding mechanism between DDX10 and Rab27b, we discovered that the DDX protein family has been affirmed to form phase-separated condensates due to their IDRs [29]. To ascertain whether this phenomenon also transpires in DDX10, we analyzed the IDR structure of DDX10 through iupred2a and PhaSePre and then found that the IDRs in DDX10 are situated in the protein sequences ranging from residue 1 to 37 and from residue 597 to 875 (Fig. 5A), and PhaSePred indicated that DDX10 exhibited a high self-phase separation and part-phase separation score (Fig. 5B and C). These results imply that DDX10 possesses the potential for undergoing phase separation. In the subsequent validation experiments, IF images showed that DDX10 forms micrometer-sized droplets, which are considered a sign of the occurrence of phase separation [30] in the nucleus of cells, and then the aggregates formed by DDX10 were disrupted by 1,6-Hex (a phase separation inhibitor) (Fig. 5D). Furthermore, we expressed DDX10-GFP (green fluorescent protein) in SCC15 cells to validate the droplet characteristic. Through live cell imaging, we dynamically observed 2 puncta fused into larger droplet (Fig. 5E). As shown in Fig. 5F and G, DDX10-GFP droplets recovered most fluorescence signals within minutes in total and subregion fluorescence recovery after photobleaching (FRAP) analysis in live cells. These results demonstrate that DDX10 proteins can undergo phase separation, and that DDX10 condensates exhibit a highly dynamic droplet formation process in OSCC cells. Previously, the binding site between DDX10 and Rab27b as predicted by AlphaFold3 was located within the IDR (Fig. 4A). Hence, we further examined whether DDX10 could interact with Rab27b to form condensation. The confocal images showed that DDX10 and Rab27b were colocalized within the nucleus and formed micrometer-sized droplet-like structures together; after the addition of 1,6-Hex or knockdown of DDX10, the formation of these droplets was markedly restrained (Fig. 5H). The above results confirm that DDX10 interacts with Rab27 within the nucleus by undergoing phase separation.

### DDX10 knockdown of OSCC restored the viability and function of T cells via exosome in vitro

**Fig. 6.**
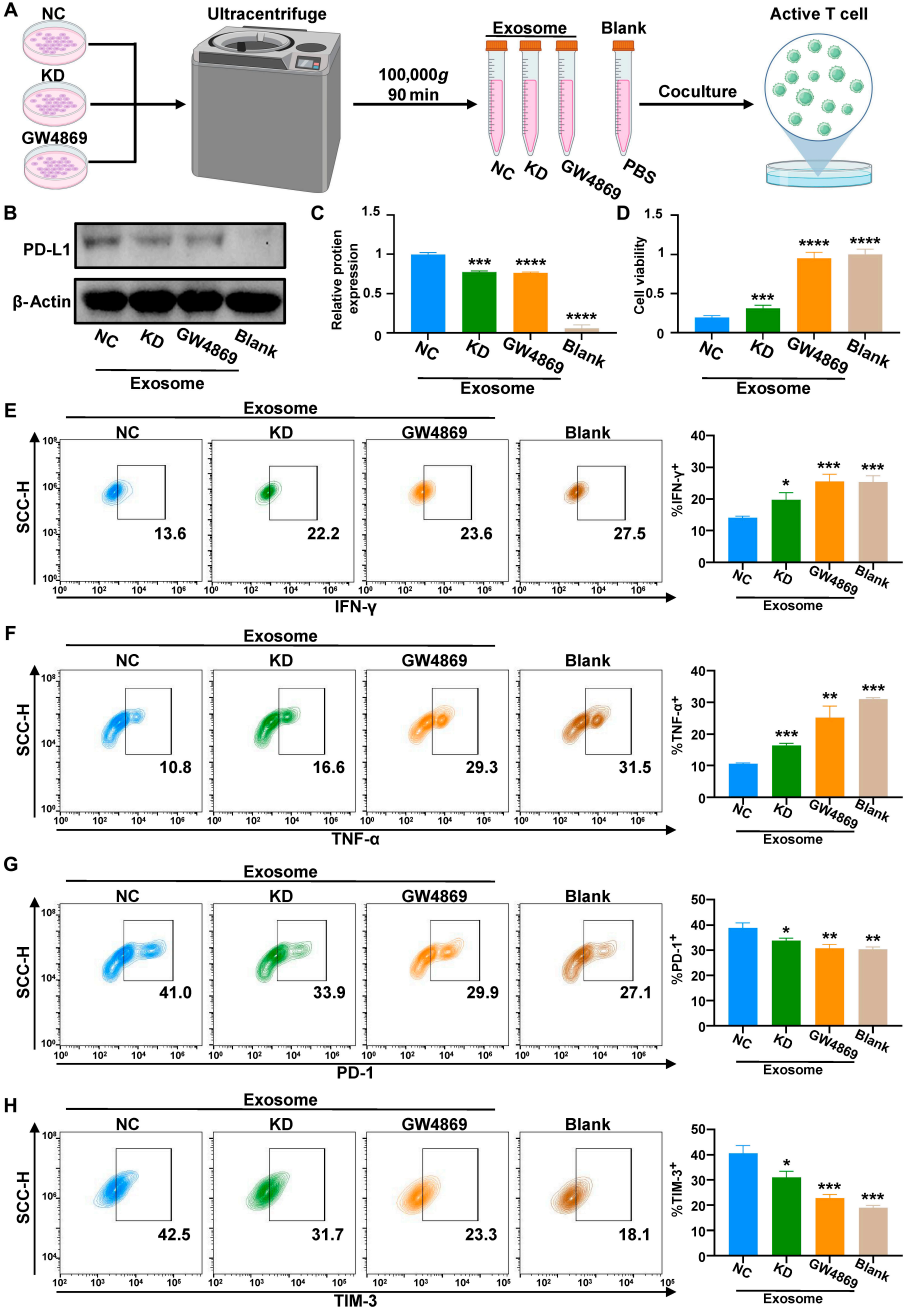
DDX10 knockdown of OSCC restored the viability and function of T cells via exosome in vitro. (A) Schematic diagram of the T cell cocultured with exosomes or PBS experimental design. (B and C) WB analysis revealed the binding level of PD-L1 to T cells cocultured under different conditions. (D) Proliferation ability of different group coculture T cells by CCK-8 assay. (E to H) Analysis of different group cocultured T cell cytokine and immune checkpoint expression by FC assays. Results are expressed as the mean ± SD from a minimum of 3 experimental replicates. *P < 0.05, **P < 0.01, ***P < 0.001, ****P < 0.0001.

Based on RNA-Seq analysis, we examined the regulatory influence of DDX10 on T cell functionality. We carried out treatment with shNC, shDDX10, and GW4869 (an exosome inhibitor) on MOC2 cells, and then purified exosomes from the culture supernatant of these cells or phosphate-buffered saline (PBS) were cocultured with activated CCL2 (Fig. 6A). Upon coculturing with exosomes, the binding of PD-L1 to T cells was significantly suppressed in the KD and GW4869 groups through WB analysis (Fig. 6B and C). The study demonstrates that tumor-derived exosomal PD-L1 specifically engages with T cell surface PD-1 receptors, consequently driving T lymphocyte exhaustion within the immunosuppressive tumor niche [31]. Consequently, we employed CCK-8 and flow cytometry (FC) assay to validate the viability and function of T cells. The former analysis demonstrated that T cell proliferation was most inhibited in the NC group, whereas T cell proliferation was progressively restored following DDX10 knockdown and subsequent GW4869 treatment (Fig. 6D). Then, FC results confirmed that the expression of cytokines tumor necrosis factor-α (TNF-α) and interferon-γ (IFN-γ) in cocultured T cells was elevated after knockdown of DDX10 in MOC2 cells, and the expression in T cells after GW4869 treatment in MOC2 cells showed no significant difference from that of T cells in the blank group (Fig. 6E and F). Conversely, the expression of immune inhibitory checkpoints PD-1 and TIM-3 in T cells was down-regulated successively after knockdown of DDX10 or GW4869 treatment in MOC2 cells, and the expression of immune inhibitory checkpoints in cocultured T cells after nature treatment was the highest (Fig. 6G and H). These results suggested that knockdown of DDX10 could restore the T cell function by inhibiting expression of exosomal PD-L1. The above findings identify that DDX10 mediated the secretion of exosomes in OSCC, thereby regulating the expression of exosomal PD-L1, which can result in T cell exhaustion.

### Knockdown of DDX10 inhibited the progression of OSCC and expression of exosomal PD-L1 in vivo

**Fig. 7.**
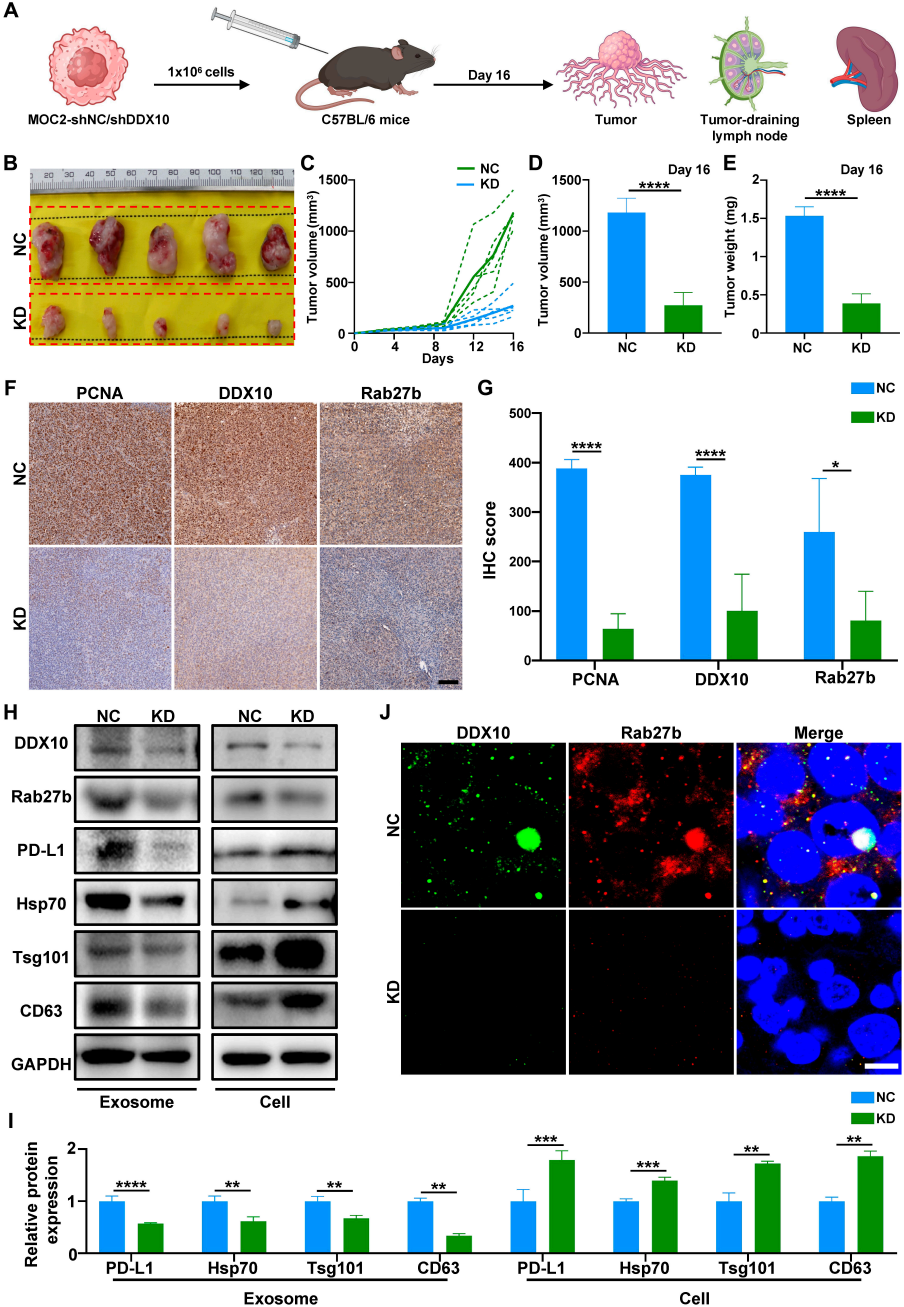
Knockdown of DDX10 can inhibit the progression of OSCC and expression of exosomal PD-L1 in vivo. (A) Schematic diagram of the vivo experimental design. (B) Comparative images of OSCC tumor between control and knockdown cohorts (*n* = 5). (C and D) Tumor volumes were measured every 3 d (C) and on final day (D). (E) Tumor weight, which was measured on final day. (F and G) Comparative IHC images and relative quantitative analysis of PCNA, DDX10, and Rab27b expressed between the NC group and the KD group. Scale bar, 100 μm. (H and I) Protein levels of DDX10, Rab27b, PD-L1, and exosomal markers were detected by WB assay in tumor cells and exosomes with or without DDX10 knockdown. (J) Representative micrographs of condensation of DDX10 and Rab27b analyzed by IF between the NC group and the KD group. Results are expressed as the mean ± SD from a minimum of 3 experimental replicates. *P < 0.05, **P < 0.01, ***P < 0.001, ****P < 0.0001.

To further confirm the promotive effect of DDX10 on OSCC development in vivo, we employed shDDX10 into MOC2 cells in vitro and injected subcutaneously into C57BL/6 mice to establish xenograft model. All mice were euthanized, and their tumors, spleens, and draining lymph nodes were harvested for further analysis at the end of the experiments (Fig. 7A). We observably perceived that the tumor volume collected from the KD group was conspicuously smaller compared to the NC group (Fig. 7B). Tumor progression in the KD cohort demonstrated marked inhibition at both singular and aggregate scales, with terminal tumor mass and volumetric parameters exhibiting statistically significant attenuation relative to the NC cohort (Fig. 7C to E), while the body weight between NC and KD groups had no significant difference (Fig. S3A). Subsequently, immunohistochemistry (IHC) analysis showed that the growth marker proliferating cell nuclear antigen (PCNA) expression was considerably lower in the KD group, while the expression level of Rab27b in the KD group declined as DDX10 was knocked down in tumor (Fig. 7F and G). Furthermore, the WB analysis of tumors indicated that the knockdown of DDX10 decreased the expression of Rab27b and exosomal PD-L1, suggesting that DDX10 regulated the expression of exosomal PD-L1 via Rab27b-mediated exosome secretion in vivo (Fig. 7H and I). To further elucidate the binding mechanism of DDX10 and Rab27b in vivo, we carried out confocal IF staining on tumor tissue sections, and the results discovered that DDX10 and Rab27b formed micrometer-sized droplet-like structures within the nucleus of OSCC cells, while the intracellular droplets were markedly reduced after knocking down DDX10, suggesting that DDX10 interacts with Rab27b within the nucleus by undergoing phase separation in vivo (Fig. 7J). Based on the aforementioned findings, we found that DDX10, as a crucial protein within OSCC, is capable of binding to Rab27b via phase separation, thereby facilitating the secretion of exosomal PD-L1 and ultimately promoting tumor progression.

### Knockdown of DDX10 facilitated T cell-mediated antitumor immunity and systemic immunity in vivo

**Fig. 8.**
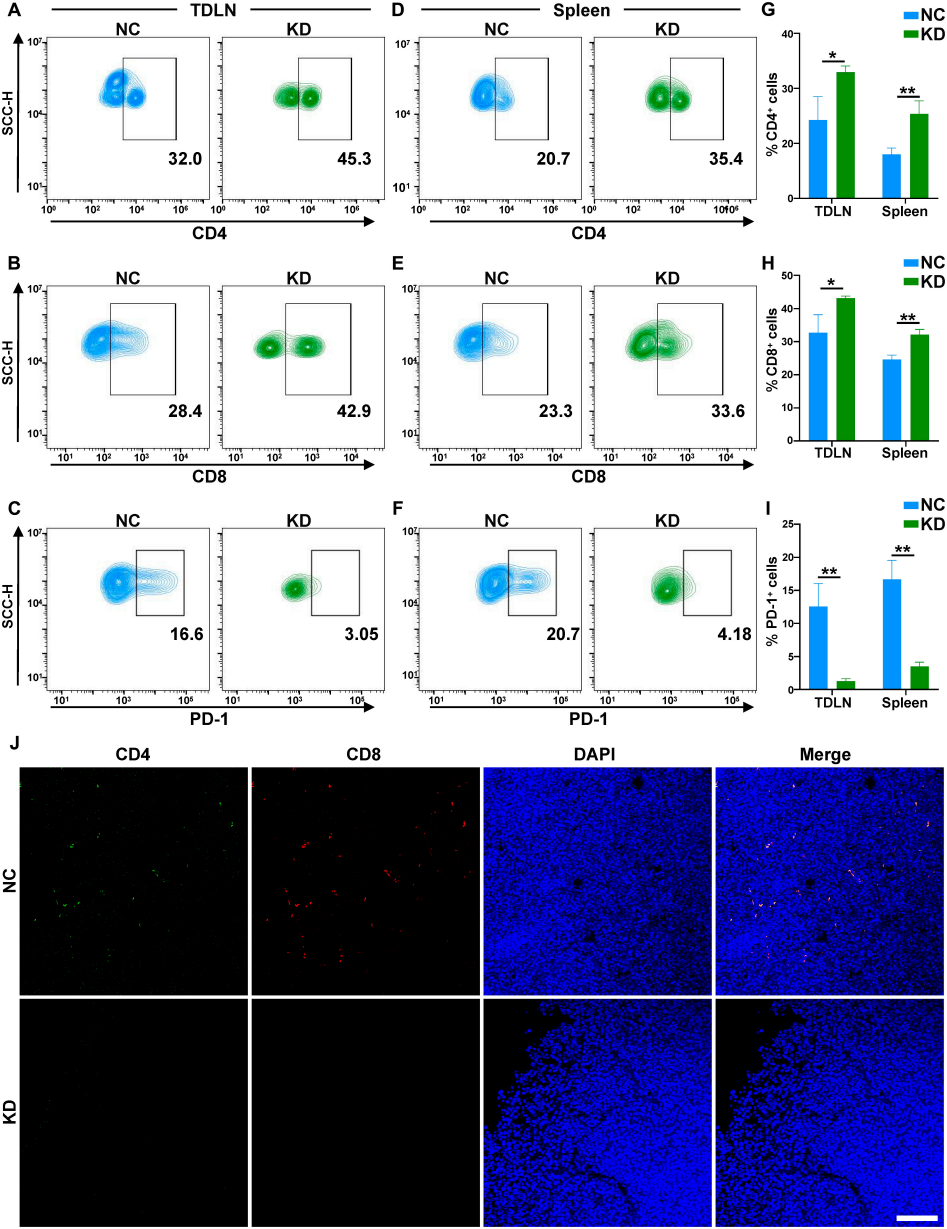
Knockdown of DDX10 facilitated T cell-mediated antitumor immunity and systemic immunity in vivo. (A to C) FC analysis showed the proportion of CD3^+^CD4^+^ T cell and CD3^+^CD8^+^ T cell and the expression of T cell PD-1 in the lymph nodes from NC and KD mice. (D to F) FC analysis revealed the proportion of CD3^+^CD4^+^ T cell and CD3^+^CD8^+^ T cell and the expression of T cell PD-1 in the spleens from NC and KD mice. (G to I) Quantitative bar charts for FC assay data analysis. (J) Representative CD4^+^ and CD8^+^ IF images of OSCC tumor with or without DDX10 knockdown. Scale bar, 100 μm. Results are expressed as the mean ± SD from a minimum of 3 experimental replicates. *P < 0.05, **P < 0.01.

Subsequently, we conducted FC analysis to explore the T cell infiltration of tumor-draining lymph nodes and spleen. The results indicated that the proportion of CD4^+^ and CD8^+^ T cells in the tumor-draining lymph nodes increased because the expression of PD-1 was down-regulated after DDX10 knockdown in vivo (Fig. 8A to C and G to I). Similarly, the proportion of CD4^+^ and CD8^+^ T cells in the spleen was augmented and PD-1 expression was diminished after DDX10 knockout in vivo (Fig. 8D to I). In addition, the confocal images demonstrated that the proportion of CD4^+^ and CD8^+^ T cells within the tumor was significantly elevated after DDX10 knockdown (Fig. 8J). Moreover, the study observed that the expression of IFN-γ was significantly up-regulated in tumors following the knockout of DDX10 via IHC assays (Fig. S3B). In conclusion, our finding confirmed that DDX10 knockdown promoted the increased infiltration of T cells by reducing their exhaustion and enhanced systemic immunity in vivo.

### DDX10 was positively correlated with Rab27b and negatively related with tumor-infiltrating T cells in OSCC tissue microarray

**Fig. 9.**
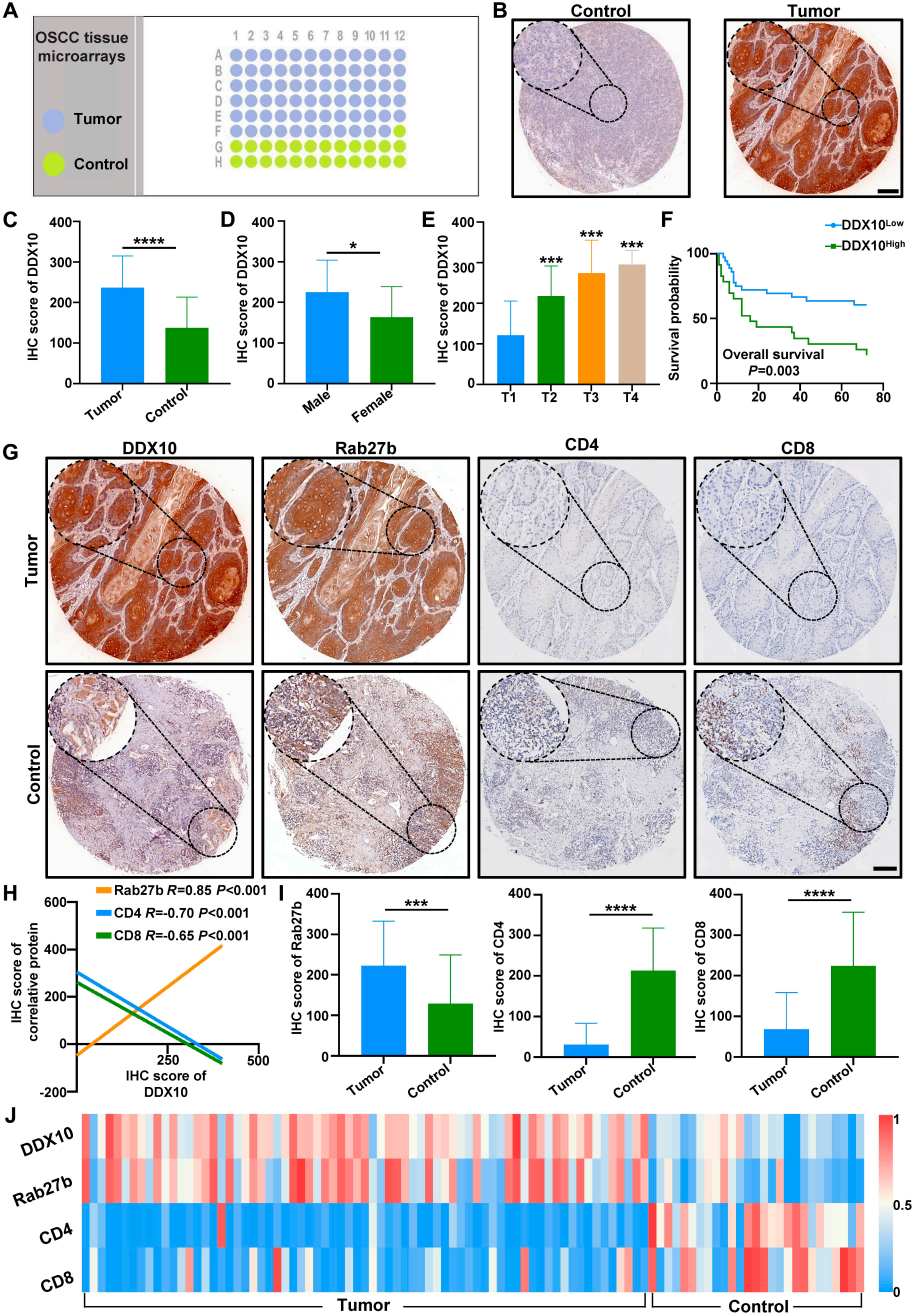
DDX10 was positively correlated with Rab27b and negatively related with tumor-infiltrating T cells in OSCC tissue microarray. (A) Paradigm of human OSCC tissue microarrays. (B and C) Comparative IHC staining images and quantitative analysis of DDX10 expression between the control group and the tumor group. Scale bar, 200 μm. (D to F) Correlation between DDX10 expression and the clinical features and prognostic outcomes in OSCC patients. (G) Representative immunohistochemical staining images of DDX10, Rab27b, CD4, and CD8 expressed in the control and tumor group. (H) Correlation analysis of DDX10 with Rab27b, CD4, and CD8 in OSCC using Pearson correlation analysis. (I) Quantitative bar charts for immunohistochemical staining scores in OSCC tissue microarrays. (J) Heatmap of expression of DDX10, Rab27b, CD4, and CD8 in OSCC tissue microarrays. Results are expressed as the mean ± SD from a minimum of 3 experimental replicates. *P < 0.05, **P < 0.01, ***P < 0.001, ****P < 0.0001.

Ultimately, tissue microarray analysis was conducted on the collected OSCC tumor tissue specimens to further validate the association of DDX10, Rab27b, and tumor-infiltrating T cells (Fig. 9A). It was revealed that DDX10 was markedly higher in the tumor group (Fig. 9B and C), especially in male patients (Fig. 9D). There is a positive correlation between the DDX10 in OSCC tumor tissues and both the clinical stage severity and prognosis, with higher expression of DDX10 indicating more advanced clinical stages and poorer prognoses (Fig. 9E and F). High expression level of Rab27b is concurrently associated with a poorer prognosis in OSCC patients (Fig. S4A). Representative IHC stain images for the control and tumor group in Fig. 9G, the expression of Rab27b was augmented in OSCC, while the infiltration of CD4^+^ and CD8^+^ T cells within the tumor was markedly reduced. The analysis of correlational relationships demonstrated a significant positive correlation between DDX10 and Rab27b, but a negative correlation with CD4^+^ and CD8^+^ T cell (Fig. 9H). This finding is further substantiated by the analyzed quantitative relationship (Fig. S3B to D). Further correlation analysis revealed Rab27b expression alongside reduced infiltration of CD4^+^ and CD8^+^ T cells in OSCC (Fig. 9I). To offer a more intuitive manifestation of the correlation among these markers, we summarized the data for each sample and then constructed a heatmap, which discovered that a high expression of DDX10 frequently led to the up-regulation of Rab27b expression, which gave rise to a reduction in the infiltration of CD4^+^ and CD8^+^ T cells in cancer (Fig. 9J), further corroborating our previous results.

## Discussion

The predominant treatment for OSCC remains surgery, chemotherapy, radiotherapy, or combination therapy, while patient survival rates have not shown marked improvement in recent years [32,33]. Targeted cancer therapy as a novel modality can compensate the limitations of the aforementioned treatment modalities [3]. Currently, research on targeted therapy for OSCC encompasses immunotherapy, gene therapy, bionanoparticle-targeted therapy, and biomarker-targeted therapy; however, their effects have not reached the ideal state [34,35]. Hence, the development of new targets remains necessary [36]. Recently, it has been verified that DDX family proteins are associated with cancer progression, and DDX10 has promotional function in cancer [15,37,38]. However, the specific biological contributions and molecular pathways of DDX10 in OSCC have not been comprehensively clarified.

In this study, DDX10 was regarded as a promising prognostic biomarker for OSCC patients. Overexpression of DDX10 was associated with aggravated clinical and pathological grade of OSCC and presented a poorer clinical prognosis in the TCGA database. We observed the marked effect of DDX10 knockdown and overexpression in proliferative, migratory, and invasive abilities of OSCC cells in vitro, suggesting that DDX10 facilitates the pathogenesis of OSCC.

To elucidate the specific mechanism of action of DDX10 in OSCC, RNA-Seq analysis demonstrated that knocking down DDX10 results in the down-regulation of the PD-L1/PD-1 immune checkpoint axis and the up-regulation of T cell signaling pathways in OSCC. The PD-L1/PD-1 immune checkpoint axis, a classic immune inhibitory signaling pathway, has been repeatedly verified to mediate tumor immune evasion, including that of OSCC, and suppresses T cell function, which is indispensable for host-mediated cancer immunosurveillance [19,39]. Currently, the studies indicate that the PD-1/PD-L1 immune inhibitory checkpoint can target PD-1 receptors on PD-L1-targeted T cells via the exosome form, thereby inhibiting immune function [28,31]. Moreover, we discovered that the down-regulation of DDX10 would lead to the down-regulation of Rab27b, which acts as a regulatory molecule for exosomes [26]. Recent studies have confirmed that tumor-derived exosomes, as a subpopulation of the tumor microenvironment, play a critical role in tumor regulation by promoting tumor cell proliferation and metastasis while suppressing intratumoral immune surveillance [40,41]. Rab27b has been shown to facilitate tumor progression by modulating exosomal release, making it a promising therapeutic target for cancer treatment [27,42]. To further validate this mechanism, we initially confirmed that DDX10 physically interacts with Rab27b and that knockdown of DDX10 suppresses the expression of Rab27b, thereby inhibiting exosome secretion and the expression of exosomal PD-L1. Subsequently, in coculture experiment, we discovered that the exosomes from DDX10-knockout cells had a decreased expression of PD-L1 and manifested a weakened inhibitory effect on T cell function and proliferation, thereby confirming the regulatory role of DDX10 on immune T cells. Upon the knockout of DDX10 in tumor xenograft, we witnessed a notable suppression of tumor growth, which was attributed to a decrease in exosomal PD-L1, resulting in weakened tumor-mediated immune suppression and an increase in T cell infiltration within the tumor, ultimately causing the inhibition of tumor progression. Ultimately, we analyzed human OSCC samples and discovered that the up-regulation of DDX10 in tumors was correlated with the deterioration of OSCC clinical grade and unfavorable prognosis for patients, along with a decrease in the infiltration of CD4^+^ and CD8^+^ T cells within tumor. Based on these findings, it can be inferred that DDX10 is capable of up-regulating Rab27b-mediated exosome secretion containing PD-L1, thereby leading to T cell exhaustion to promote OSCC progression.

Phase separation, a phenomenon where biological molecules interact with each other, forms droplet-like condensation within cells to act as membrane-less organelles and support the progression of life activities [43]. The studies have confirmed that proteins of the DDX family can form phase-separated condensation, where the condensation exerts a positive regulatory effect on tumors [4,29]. Based on this, we predicted that DDX10 possesses an IDR structure, a low-complexity and highly active structure that promotes the formation of phase separation [44], and has the potential to undergo phase separation. Subsequently, we found that DDX10 formed typical droplet-like phase separation structures, which were blocked by the phase separation inhibitor 1,6-Hex within the nucleus of OSCC cells. Moreover, we found the puncta fusion and FRAP in live SCC15 cells, demonstrating that DDX10 phase-separated condensates exhibit a highly dynamic droplet formation process in OSCC cells. Then, we labeled DDX10 and Rab27b with fluorescence and observed that DDX10 and Rab27b bound to each other to form droplet-like structures in vitro and in vivo. After treatment with 1,6-Hex or knockout of DDX10, these droplet-like structures also dissipated. These results indicate that DDX10 can interact with Rab27b by undergoing phase separation.

**Fig. 10.**
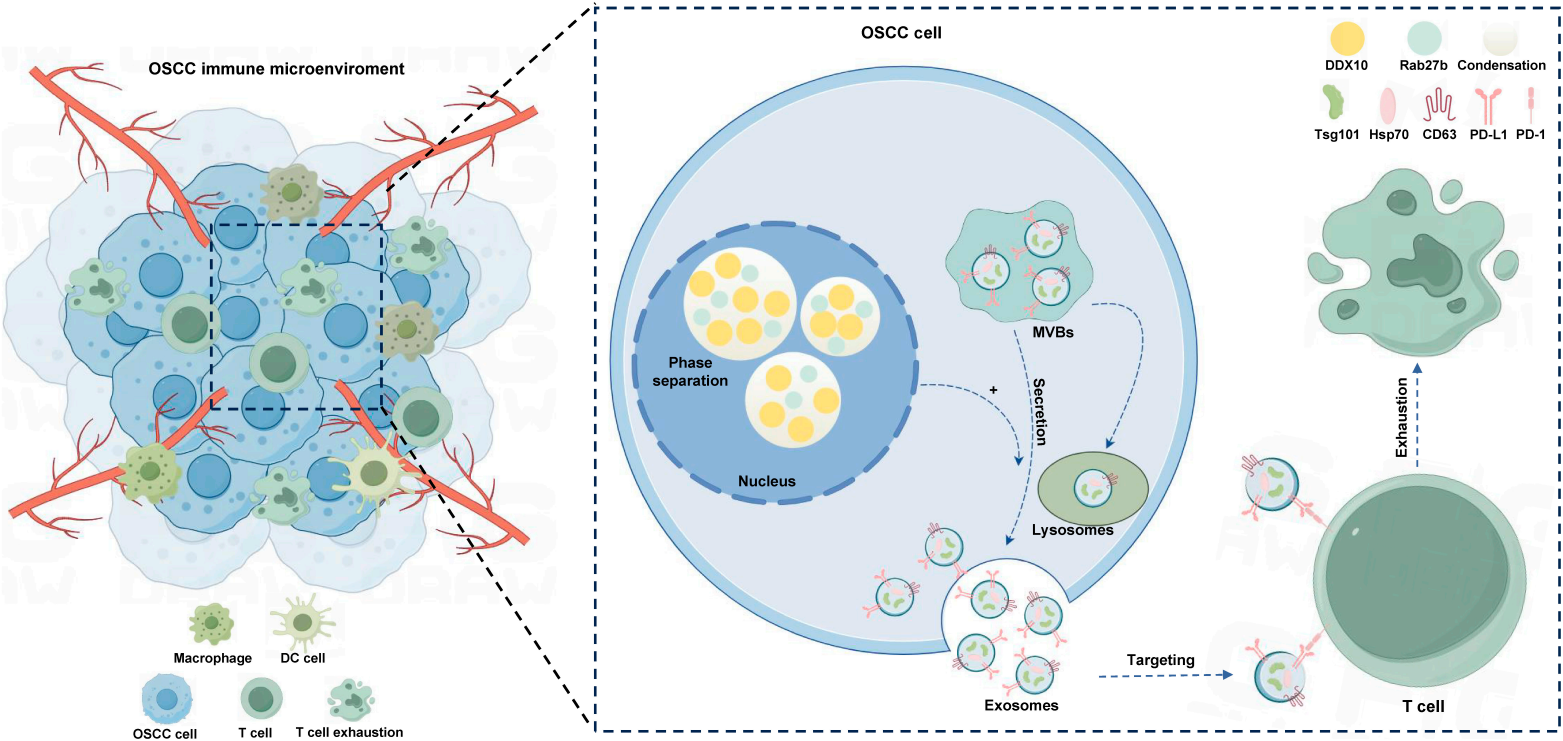
A diagrammatic representation outlining the roles of DDX10 in OSCC.

In summary, we discovered that DDX10 was overexpressed in OSCC, which was correlated with a poor clinicopathological grade and adverse clinical prognosis. DDX10 can facilitate the progression of the OSCC via the formation of bio-condensation with Rab27b. Although the exact and further mechanisms by which DDX10 influences the progression of OSCC require deeper studies, our studies show that DDX10 is capable of up-regulating Rab27b via phase separation, thereby promoting the expression of exosomal PD-L1, thus suppressing the infiltration of T cells within the OSCC immune microenvironment, ultimately facilitating the progression of OSCC (Fig. 10). This will provide a feasible target for targeted therapy and immunotherapy of OSCC, thereby enhancing the therapeutic effect of tumor treatment. Taken together, this investigation revealed DDX10 as a novel therapeutic target in OSCC, demonstrating dual oncogenic effects by enhancing malignant progression while impairing T cell-mediated antitumor immunity. This will provide a feasible biomarker for targeted therapy and immunotherapy of OSCC, thereby improving the effect of tumor treatment through a dual approach.

## Materials and Methods

### TCGA data analysis

RNA-Seq data in HNSC and OSCC were thoroughly investigated using datasets from TCGA. Specifically, the expression of DDX10 with corresponding clinical data of HNSC and OSCC patients and immune infiltration in OSCC was retrieved from the Genomic Data Commons Data Portal (https://www.kegg.jp/kegg/pathway.html).

### Patient cohort and human OSCC tissue microarrays

Clinical specimens were sourced from Southern Medical University’s Stomatology Hospital and Xiangya School of Medicine over an 8-year period spanning 2012 to 2020. All patients received an OSCC diagnosis from the hospital’s Department of Pathology. Prior to surgery, informed consent was obtained from each patient. Follow-up was conducted through phone calls or in-person visits until the conclusion of the study or the patient’s death. The concluding clinical assessments were completed in October 2020, exhibiting a median observation period of 37 months (1 to 96 months). Neoplastic specimens underwent standardized paraffin embedding protocols with 12- to 16-h fixation intervals. Initially, the samples were stained with hematoxylin and eosin (H&E), and a board-certified pathologist identified and annotated representative tumor areas in each OSCC specimen. The tissue arrays were constructed using 1.5-mm-diameter tissue cores extracted from predefined zones, containing 56 primary OSCC tissues and 20 control tissues. Survival analysis was conducted using data from 54 OSCC patients.

### Cell culture and mice

The mouse and human OSCC cell lines were acquired from Fu Heng Biology (Shanghai, China) and preserved in our laboratory, which were cultured in a CO_2_ incubator at 37 °C and 5% CO_2_ in RPMI 1640 supplemented with 10% fetal bovine serum (FBS) (Gibco) and 100 U/ml penicillin–streptomycin (Gibco). CTLL-2 (mouse T cell line) was acquired from Pricella (Wuhan, China), which was cultured in a CO_2_ incubator at 37 °C and 5% CO_2_ in the specific medium. Six-week-old C57BL/6 mice (female, 18 to 20 g) were purchased from Guangzhou Ruige Life Science Company (Guangzhou, China). All mice were raised in a specific pathogen-free environment at the Animal Experiment Center of Southern Medical University.

### Cell transfection

The synthesis of small interfering RNA (siRNA) targeting human DDX10 was conducted by Songgon Biotech (Shanghai, China), and the transfection was carried out using Transfection Reagent A (Songgon Biotech, Shanghai, China). The synthesis of plasmid containing GFP-DDX10 was conducted by Synbio Technologies (Suzhou, China). Short hairpin RNA (shRNA) targeting mouse DDX10 was inserted into the pLKO.1-copGFP-PURO-puromycin lentiviral vector (Synbio Technologies, Suzhou, China). The transfection efficiency was confirmed through WB assay and IF staining. siRNA target sequences for DDX10 were si-DDX10#1: CCGAUAAAGUAAUUGAGCCAATT. shRNA target sequences for DDX10 were sh-DDX10#2: ATGTGAGCAAGTTACCTATTA. The sequences for the siRNAs, plasmid, and shDDX10 utilized in this investigation are shown in Table S1.

### CCK-8 assay

CCK-8 kit (Epizyme, Shanghai, China) was used to measure the proliferation of SCC15 and CTLL-2 with or without pretreatment. These cells placed in 96-well plates were cultured for 24, 48, and 72 h. Optical density at 450 nm (OD_450_) was quantified using an MD SpectraMAX microplate reader following 2-h incubation period. Ultimately, the extreme values from both ends were excluded to calculate the average. Each experiment included at least 3 replicates.

### Transwell experiment

SCC15 cells with or without pretreatment were prepared in 200 μl of serum-deprived Dulbecco’s modified Eagle’s medium (DMEM) and loaded into Transwell inserts (353097, BD Falcon, NY, USA), and the basal chambers received 600 μl of DMEM supplemented with 20% FBS (Gibco). For cell invasion evaluation using Transwell systems, Matrigel matrix (50 μl, 1 mg/ml, Beijing Solarbio Science & Technology Co) was evenly applied to the upper chamber. Following 48-h incubation, the cells adherent to the basolateral compartment were immobilized using 4% paraformaldehyde (15 min) and subsequently subjected to 0.1% crystal violet solution staining (8 min).

### Wound healing assay

Transfected cells and natural control cells were placed in 6-well plates and grown until they reached 80% to 90% confluence. Following medium aspiration, cellular monolayers underwent gentle hydration through PBS application (100-μl volume) to maintain integrity. Next, a scratch was created in the cell monolayer using a 1,000-μl pipette tip. After washing the plate with PBS, 2 ml of serum-deprived medium was dispensed into each well, and the plate was then returned to the incubator for further cultivation. Ultimately, microscopic images of the wound areas were acquired at 0, 8, 24, and 48 h post-scratch.

### RNA-Seq

RNA-Seq was conducted by Novo Tech using cell samples from both the control group and the DDX10 knockdown group. Both SCC15 cells (1 × 10^5^) were cultured in 6-well plates and then lysed and prepared into a standard sample. The extracted samples underwent quality control for total RNA, and sequencing was performed on an Illumina NovaSeq platform, followed by data analysis and the preparation of analysis result files.

### Western blot

Lysis of the cell or exosome samples was performed using sodium dodecyl sulfate (SDS) sample buffer supplemented with protease and phosphatase inhibitors, followed by incubation at 95 °C for 10 min. The supernatant was then loaded onto a 10% SDS-polyacrylamide gel (Beyotime) and separated by electrophoresis at 65 V for 30 min, subsequently increasing to 115 V for 80 min. Subsequently, electrophoretic transfer was performed at 220 mA for a specified duration in an ice bath. To block nonspecific binding sites, the polyvinylidene difluoride (PVDF) membrane was treated with a 5% bovine serum albumin solution for 1 h at room temperature, followed by overnight incubation with primary antibodies at 4 °C. The next day, after washing the membrane, it was incubated with secondary antibodies (Proteintech) for 60 min at room temperature. The blot was subsequently detected via an enhanced chemiluminescence detection kit (Biosharp, Chongqing, China). Anti-DDX10 (Proteintech, 17857-1-AP), anti-Rab27b (Proteintech, 13412-1-AP), anti-PD-L1 (Proteintech, 17952-1-AP), anti-Hsp70 (Abcam, ab181606), anti-Tsg101(Abcam, ab125011), anti-CD63 (Abcam, ab134045), anti-GAPDH (Proteintech, 60004-1-Ig), and anti-β-actin (Proteintech, 66009-1-Ig) antibodies were used for Western blot, where the anti-GAPDH or anti-β-actin antibody served as a loading control.

### Prediction for biomolecular interactions and potential of phase separation

The interaction between DDX10 and Rab27b was predicted with AlphaFold3 (https://alphafoldserver.com/), an accurate structure prediction tool for biomolecular interactions [45]. The structural analysis and figures were prepared using PyMOL. The IDR structure of DDX10 was analyzed via iupred2a (https://iupred2a.elte.hu/plot), and the potential of DDX10 undergoing phase separation was predicted with PhaSePred (http://predict.phasep.pro/), a comprehensive PS predictor [46].

### Fluorescence recovery after photobleaching

FRAP experiments were performed on a confocal microscope (Olympus FV4000). Droplets were photobleached with a 488-nm laser pulse (40% intensity, 1.5 s). Fluorescence recovery was monitored every 3 s for 180 s immediately after photobleaching. The fluorescence intensity and the images of the bleached region, reference region, and background region were recorded by the FRAP module in the cellsens software.

### Co-IP assay

The physical association between DDX10 and Rab27b was examined using an IP/Co-IP Kit (KTD104-CN, Abbkine, Wuhan, China). Following lysis of the cells, the protein samples were incubated with A/G magnetic beads that had been preincubated with the primary antibody, under gentle agitation at 4 °C for an extended period. Following the incubation period, the magnetic beads were separated with a magnetized rack, and the supernatant was carefully aspirated. To eliminate nonspecifically bound proteins, the immunocomplexes were washed with precooled lysis buffer that did not contain protease inhibitors, and then eluted from the magnetic beads by incubation at 100 °C for 10 min in 1× SDS loading buffer. The final samples were identified through WB assay.

### Isolation and characterization of exosomes

The supernatants from cells in serum-deprived medium were collected and centrifuged at 300*g*/10 min to wipe off the cells and other large particulate impurities, followed by centrifugation at 10,000*g*/35 min to wipe off large vacuoles. Exosomes were isolated through ultracentrifugation at 100,000*g* and then purified with PBS. This was followed by an additional centrifugation at 100,000*g* for 90 min. The pellet obtained after centrifugation, containing the exosomes, was resuspended in PBS and stored at −80 °C until further use. The total protein concentrations of the exosomes were measured using a BCA Protein Assay kit (Epizyme, Shanghai, China). The size distribution of the exosomes was characterized by dynamic light scattering, and their morphology was assessed using TEM. WB assay analyzed the characteristics of exosome surface marker proteins Hsp70, TSG101, and CD63.

### IF in vitro

The IF of cell climbing sheets was performed as described previously [47]. DDX10 (Proteintech, 17857-1-AP) and Rab27b (Proteintech, 13412-1-AP) were used as primary antibodies for our experiment. Laser confocal microscope inspection (Leica SP8) represented the IF images. The fusion of condensation in live SCC15 was monitored every 8 s for 300 s by a confocal microscope (Olympus FV4000).

### Exosome coculture with CTLL-2 (mice T cell) in vitro

CTLL-2 was incubated with anti-CD3 (1.5 μg/ml, BD Biosciences) and anti-CD28 (1 μg/ml, BD Biosciences) for 48 h to be activated. CTLL-2 (2 × 10^6^/well) were divided into four groups and seeded into 6-well plates. Three groups were cocultured with exosomes derived from the same number of donor cells, which had been transfected with either shNC, shDDX10, or pretreated with GW4869 prior. The remaining group was cocultured with PBS as a blank control. After coincubation for 48 h, cocultured and blank CTLL-2 were collected for Western blot and FC analysis.

### Animal assays

The animal study was performed in compliance with the ARRIVE (Animal Research: Reporting of In Vivo Experiments) guidelines, and ethical approval was obtained from the Institutional Review Board (NYKO-LAEC-2024-025). A total of 1 × 10^6^ MOC2 cells with or without knockdown of DDX10 were injected subcutaneously into C57BL/6 mice (*n* = 5). On the 16th day, euthanasia was performed on the mice using an overdose of pentobarbital anesthetic. The tumor tissues were subsequently harvested for FC analysis, measured for weight, paraffin-embedded overnight, and cryopreserved at −80 °C. Tumor dimensions were measured with a micrometer caliper, and murine body weights were monitored every other day.

### FC analysis

Tissue FC was used to identify immune cell populations in spleens and tumor-draining lymph nodes. The single-cell suspension was prepared following the methodology described in our previous study [48]. Subsequently, the cells were stained with the following antibodies: CD45 [allophycocyanin (APC)-Cy7; clone 30-F11], CD3 (BV510, clone 145-2c11), CD8a (BB515; clone 53–6.7), CD4 (BB700; clone RM4-5), and PD-1 [phycoerythrin (PE)/Cyanine7, clone RMPI-3]. CTLL-2 with or without coculture with exosome cytometry was used to detect immune cell function. After coculturing, cells were restimulated with Cell Stimulation Cocktail (MultiScience, Hangzhou, China) for 4 to 6 h and then fixed and permeabilized with Fixation & Permeabilization Kit (MultiScience, Hangzhou, China). This single-cell suspension was stained with the following antibodies: TNF-α (APC, clone MP6-XT22), IFN-γ (PE, clone XMG1.2), TIM-3 (Alexa Fluro 547, clone RMT3-23), and PD-1 (PE/Cyanine7, clone RMPI-3). Final sample analysis was conducted with Beckman CytoFLEX flow cytometer (Brea, California, USA), followed by data processing using FlowJo software (TreeStar, BD, New Jersey, USA).

### IF and IHC in vivo

Deparaffinization and antigen retrieval procedures were performed on paraffin-embedded histological specimens. The IHC primary antibodies were DDX10 (Proteintech, 17857-1-AP), Rab27b (Proteintech, 13412-1-AP), IFN-γ (Affinity DF6045), and PCNA (Cell Signaling Technology, 13,110), and digital pathology scanner (Leica) represented the images. IF primary antibodies were DDX10 (Proteintech, 17857-1-AP), Rab27b (Proteintech, 13412-1-AP), CD4 (Cell Signaling Technology, D7D2Z), and CD8 (Cell Signaling Technology, D4W2Z). Laser confocal microscope (Leica SP8) represented IF images.

### Statistical analysis

Statistical analyses were performed with GraphPad Prism v9.0 (GraphPad Software, La Jolla, CA) and SPSS 20.0 (IBM Corp., Armonk, NY) on Windows. Statistical analyses were performed to assess significant differences using normality and lognormality tests, chi-square tests, unpaired *t* tests, and Pearson correlation analysis. Each experiment was conducted in triplicate, independently repeated. Both univariate and multivariate approaches were employed for survival analysis using SPSS v20.0 (IBM Corp., NY). Optimal cutoff values for DDX10 expression levels were identified following methodologies from existing study [49]. Results are expressed as the mean ± SD from a minimum of 3 experimental replicates (*P < 0.05; **P < 0.01; ***P < 0.001; ****P < 0.0001; ns, not significant).

## Data Availability

All datasets generated and analyzed throughout this study are accessible through the corresponding author with reasonable request.
