# Supplementary material for: DDX10 Exacerbates Exosomal PD-L1-Dependent T Cell Exhaustion via Phase Separation of Rab27b in Oral Squamous Cell Carcinoma
Source: Research (Wash D C). 2025 May 9;8:0697. doi: 10.34133/research.0697 (PMC12063704; doi:10.34133/research.0697)
Supplement: Supplementary 1 — Table S1 Figs. S1 to S4 [file research.0697.f1.docx]

**Supporting Information**

**DDX10** **Exacerbates Exosomal PD-L1 Dependent T Cell Exhaustion via** **Phase Separation of** **Rab27b in Oral Squamous Cell Carcinoma**

Bowen Li ^1^, Hao Cui ^1^, Wei Liu ^1^, Zhou Lan ^1^, Chang Liu ^1^, Yumiao Yang ^1^, Yuyue Zhao ^1^, Zhen Tian ^1^, Hao Chen ^1^, Guangtao Yu ^1*^

^1^Stomatological Hospital, School of Stomatology, Southern Medical University, Guangzhou, Guangdong, 510280, China.

**Table 2. Target sequences for siRNA/shRNA/Plasmids**

| **Category** | **Gene** | **Species** | **Target sequences (5’→3’)** |
| --- | --- | --- | --- |
| siRNA | siNC | Human | UUCUCCGAACGUGUCACGUTT |
|  | siDDX10-1 | Human | CCGAUAAAGUAAUUGAGCCAATT |
|  | siDDX10-2 | Human | CCAGUGCUGGAAGCCUUAUAUTT |
|  | siDDX10-3 | Human | UACUCUUUGCUACUGAUAUUGTT |
| shRNA | shNC | Mouse | TTCTCCGAACGTGTCACGTTTCAAGAGAACGTGACACGTTCGGAGAATTTTTT |
|  | shDDX10-1 | Mouse | GAGGATGCCAACACGTATATT |
|  | shDDX10-2 | Mouse | ATGTGAGCAAGTTACCTATTA |
|  | shDDX10-3 | Mouse | GCACAGTACCGCTTGGTAACA |
| Plasmids | pCMV-EGFP-DDX10-WT | Human | Synbio |

**
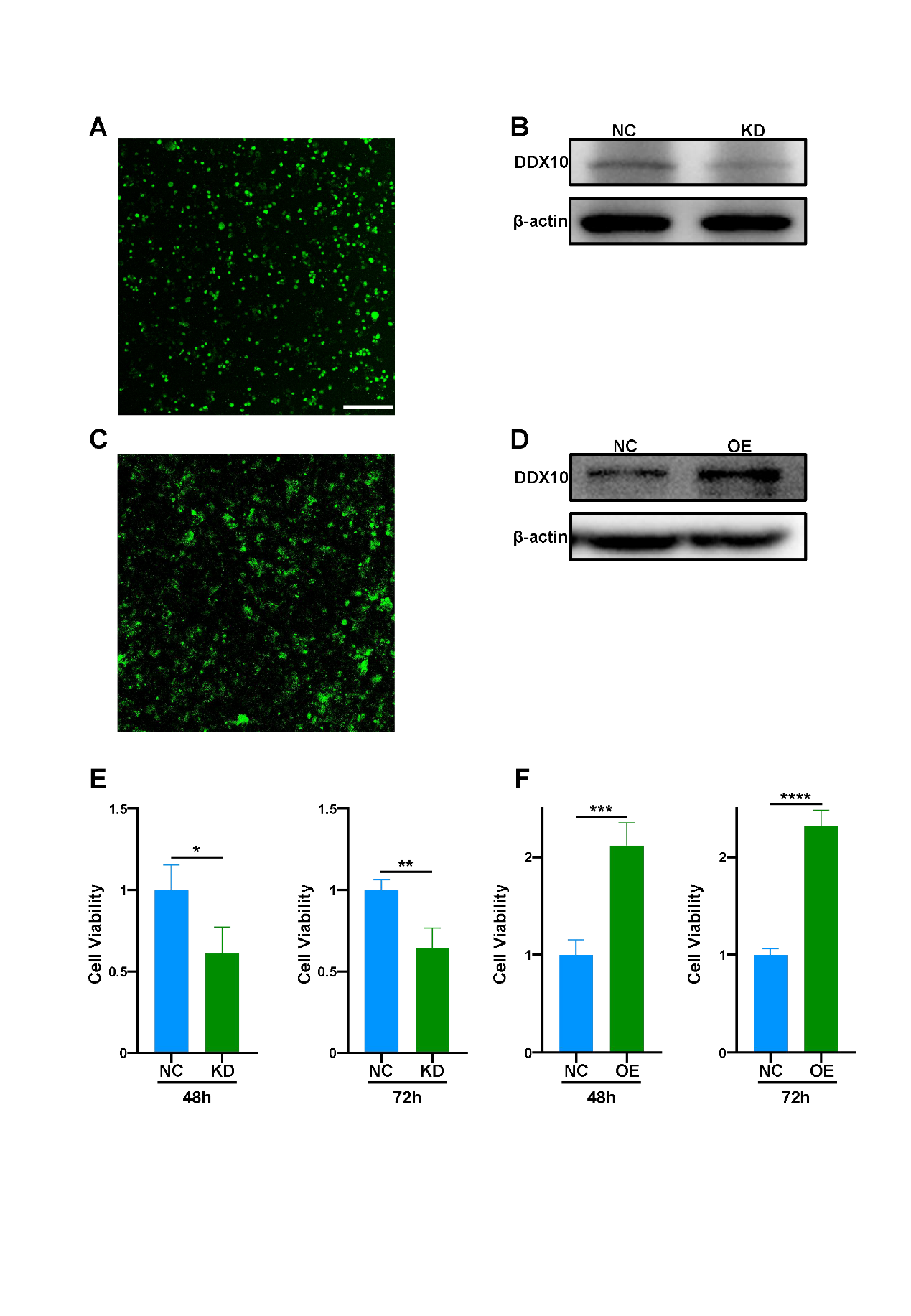
**

**Fig S1.** The transfection efficiency and knockdown effect of siDDX10. (A and C) The representative micrographs of transfection efficiency analyzed by immunofluorescence in SCC15 cells. Scale bar: 200μm. (B and D) The transfection effect of DDX10 analyzed by Western blot in SCC15 cells. (E-F) Impact of DDX10 knockdown and overexpression on the proliferative capacity of SCC15 cells by CCK-8 assay in 48h and 72h. ns: not significant, **p*<0.05, ***p*<0.01, ****p*<0.001, *****p*<0.0001.

**
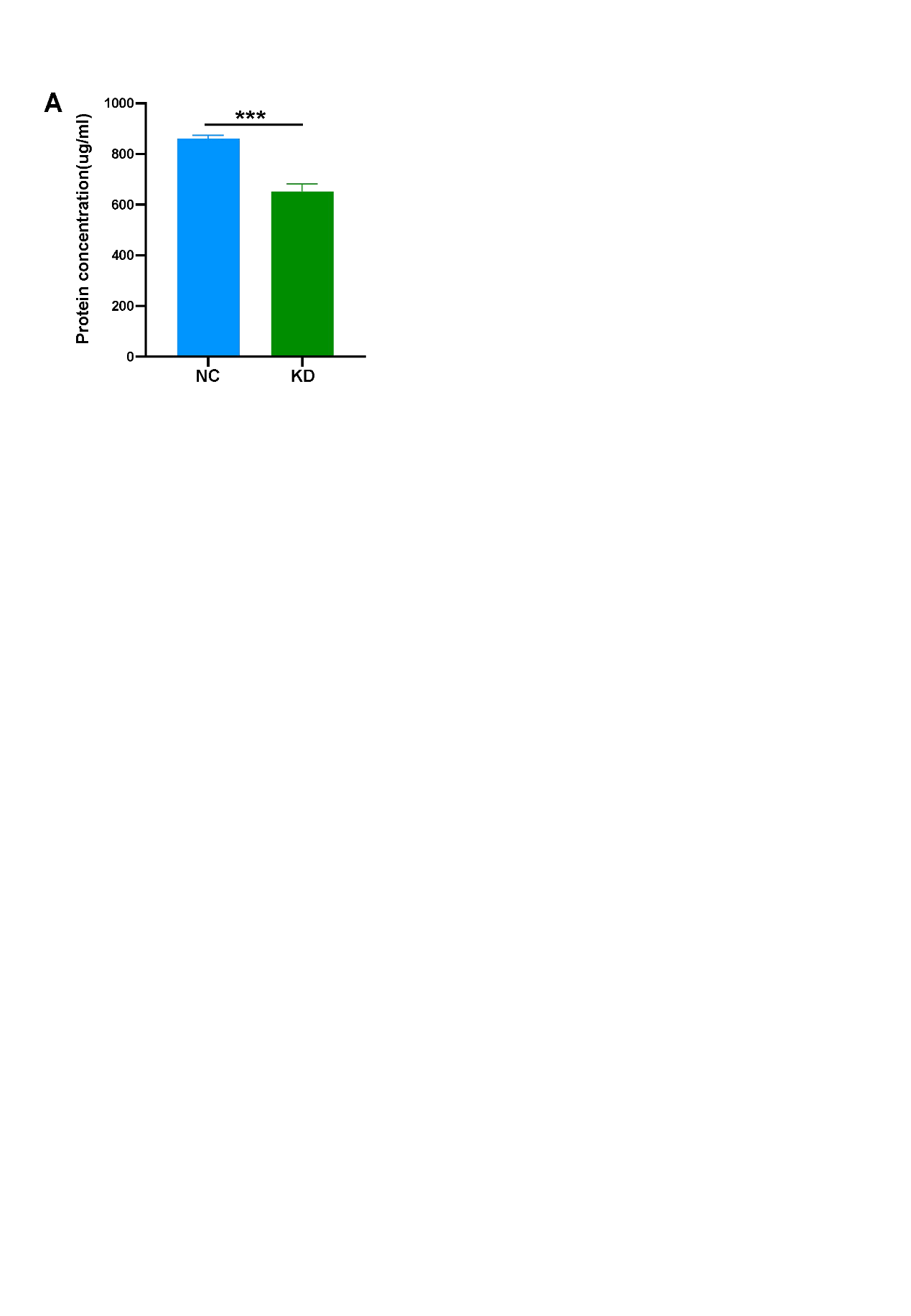
Fig. S2.** (A) The BCA concentration analysis of total exosome proteins from SCC15 cells with or without knockdown of DDX10. Data are presented as mean ± SD of three independent experiments. ns: not significant, **p*<0.05, ***p*<0.01, ****p*<0.001, *****p*<0.0001.

**
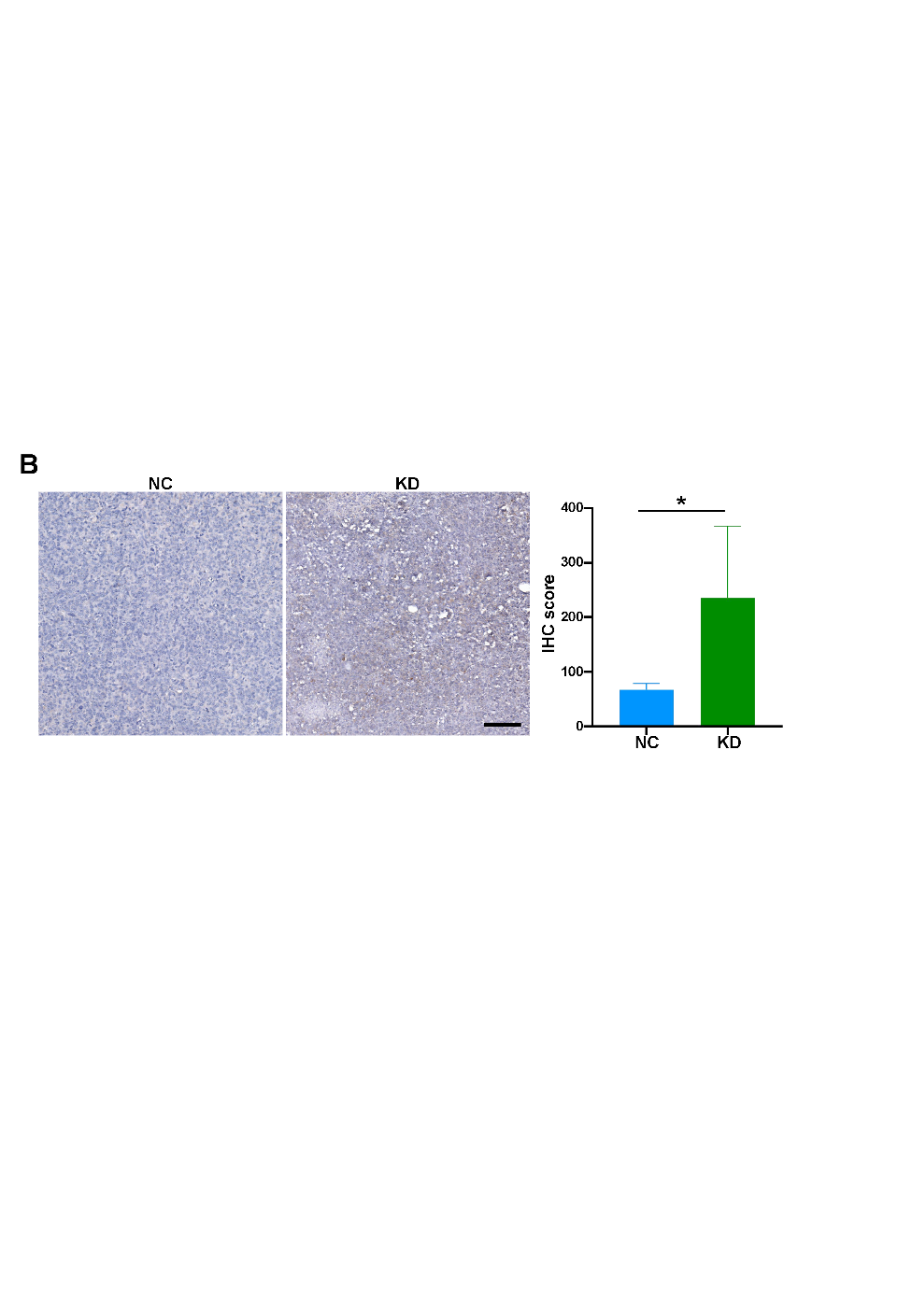

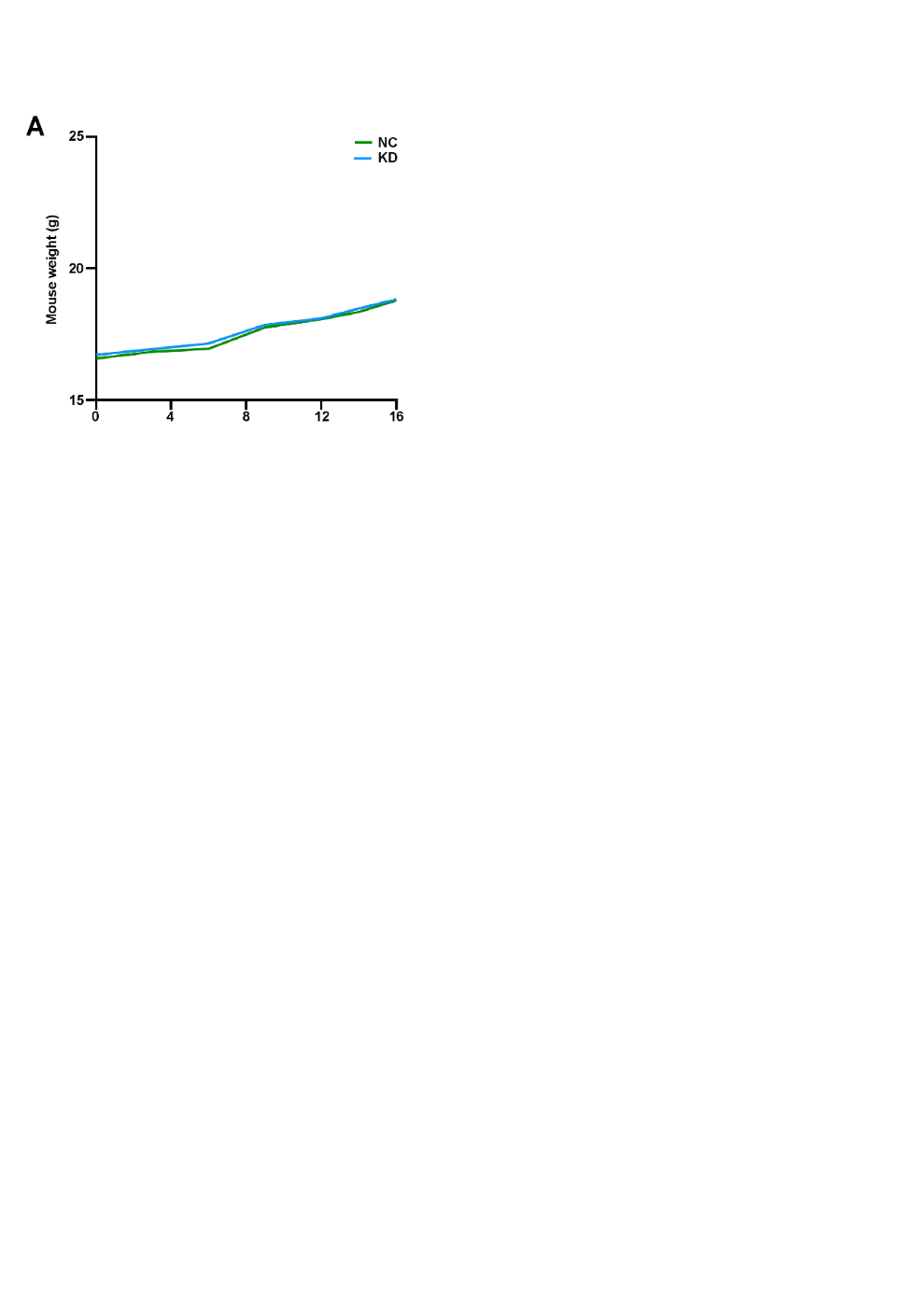
**

**Days**

**Fig. S3.** (A) The total weight of mice treated with xenograft tumors with or without knockdown of DDX10. (B) Comparative IHC images and relative quantitative analysis of IFN-γ expressed in OSCC tumor between NC group and KD group. Scale bar = 100 μm. **p*<0.05.

**
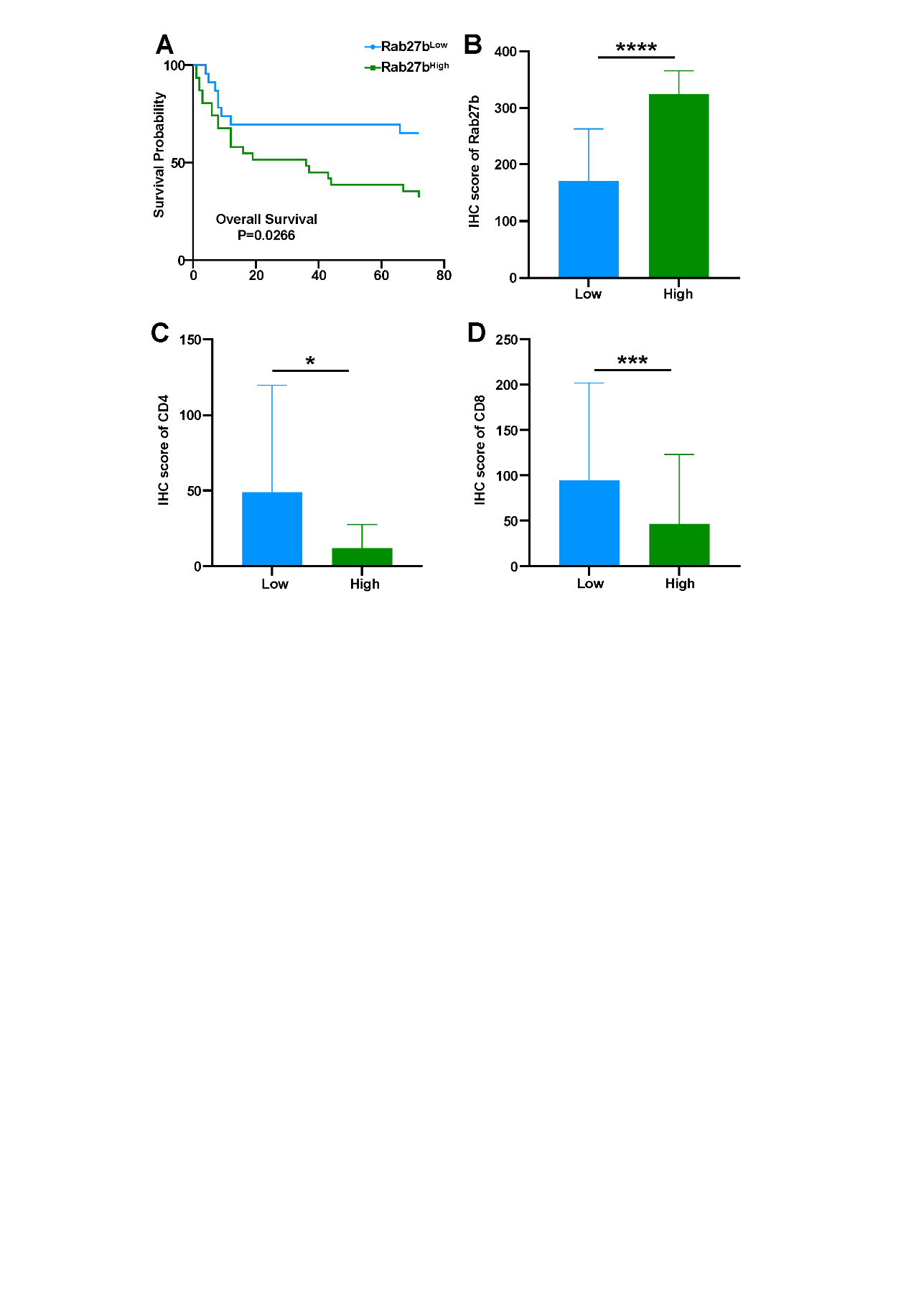
**

**Fig. S4.** The expression difference of downstream protein between high and low expression of DDX10. (A) The correlation between Rab27b expression and the prognostic outcomes in OSCC patients. (B) The expression difference of Rab27b between high and low expression of DDX10 in human OSCC samples. (C) The expression difference of CD4 between high and low expression of DDX10 in human OSCC samples. (D) The expression difference of CD8 between high and low expression of DDX10 in human OSCC samples. Data are presented as mean ± SD of three independent experiments. ns: not significant, **p*<0.05, ***p*<0.01, ****p*<0.001, *****p*<0.0001.
